# Supplementary material for: Identification of a Novel Gene Signature with DDR and EMT Difunctionalities for Predicting Prognosis, Immune Activity, and Drug Response in Breast Cancer
Source: Int J Environ Res Public Health. 2023 Jan 10;20(2):1221. doi: 10.3390/ijerph20021221 (PMC9859620; doi:10.3390/ijerph20021221)
Supplement: Supplementary file 1 [file ijerph-20-01221-s001.zip › Supplementary file S10.pdf]

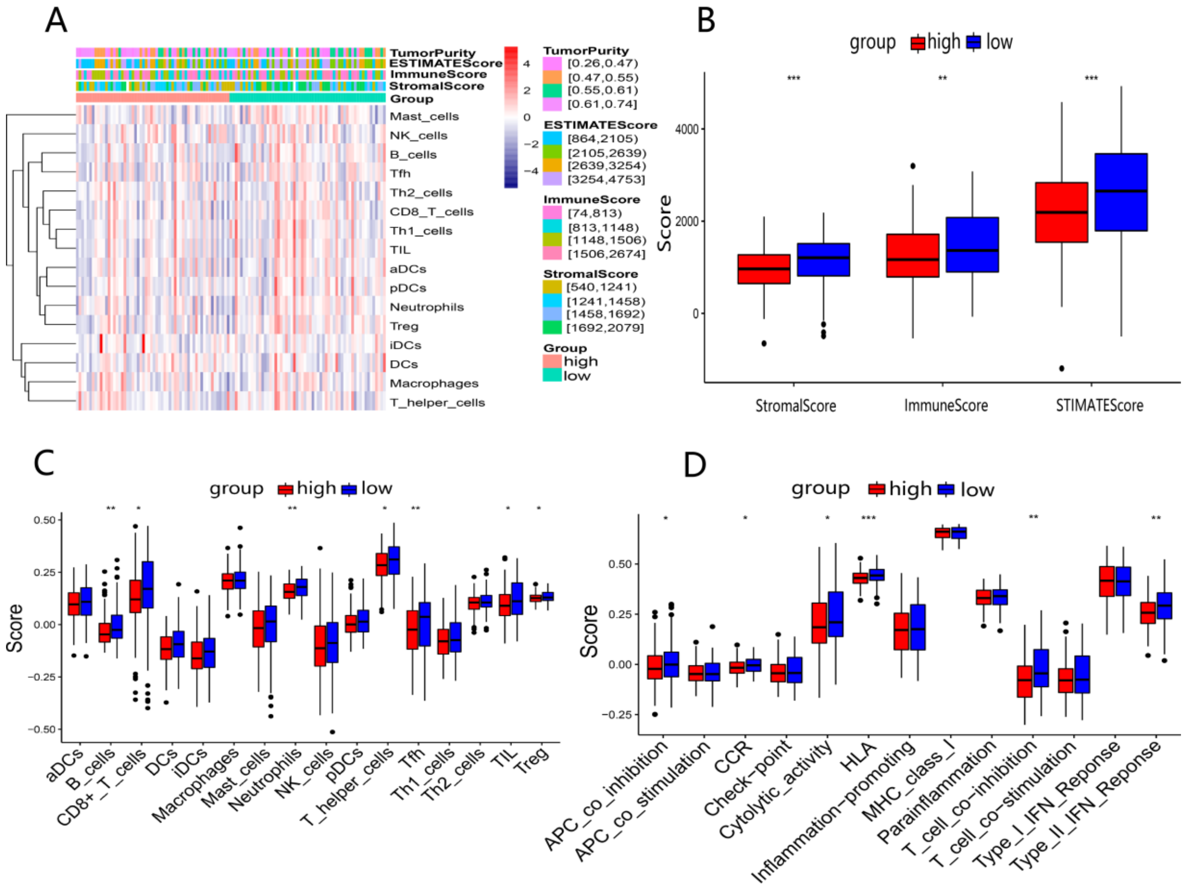

Figure S1. Analysis of immune activity in two risk groups in GSE20685-BRCA. **(A)** Comparison of enrichment levels of immune cells and types between two risk groups. **(B)** Box plots used to compare the immune score, stromal score, and ESTIMATE score for two risk groups. **(C,D)** Comparison of enrichment scores for 16 immune cells and 13 immune-related pathways between two risk groups. * $p < 0.05$; ** $p < 0.01$; *** $p < 0.001$.

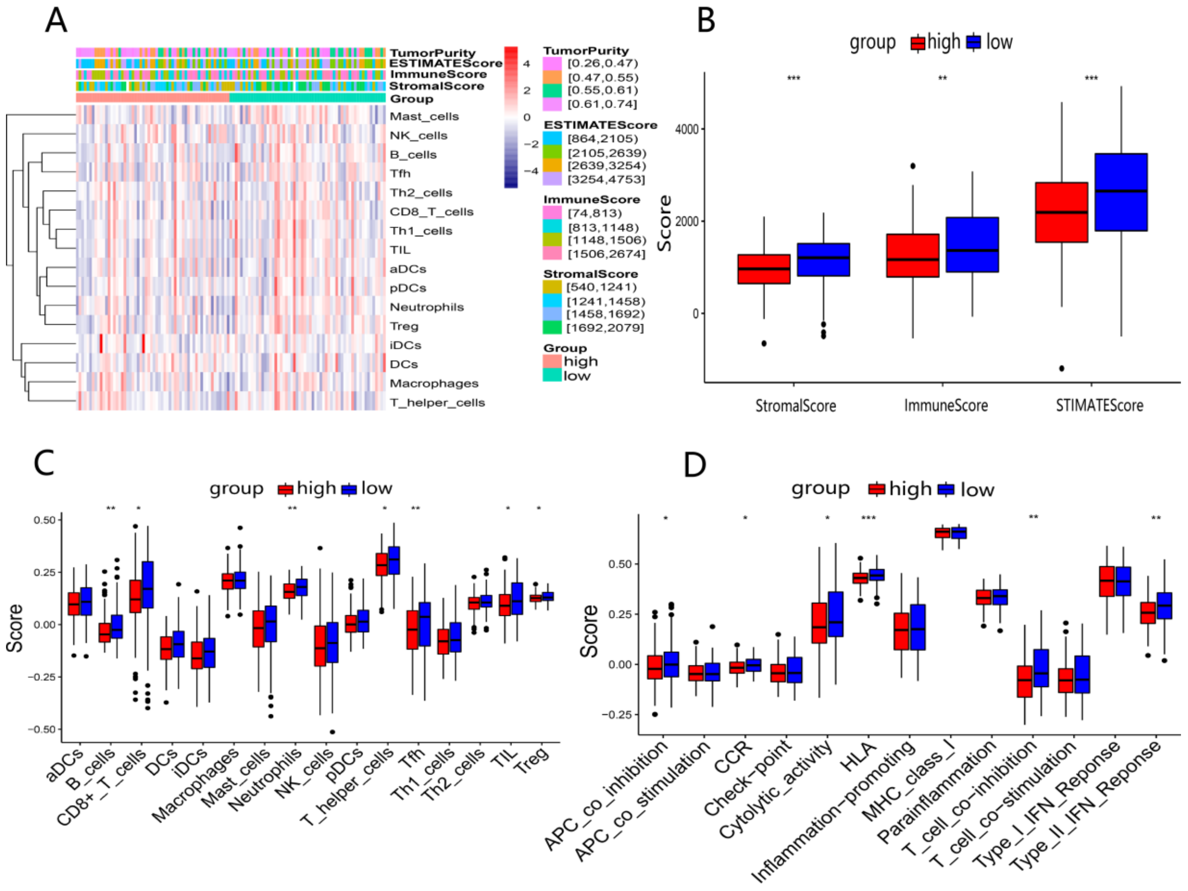

Figure S2. Analysis of immune activity in two risk groups in GSE88770-BRCA. **(A)** Comparison of enrichment levels of immune cells and types between two risk groups. (**B**) Box plots used to compare the immune score, stromal score, and ESTIMATE score for two risk groups. **(C,D)** Comparison of enrichment scores for 16 immune cells and 13 immune-related pathways between two risk groups. * $p < 0.05$; ** $p < 0.01$; *** $p < 0.001$.

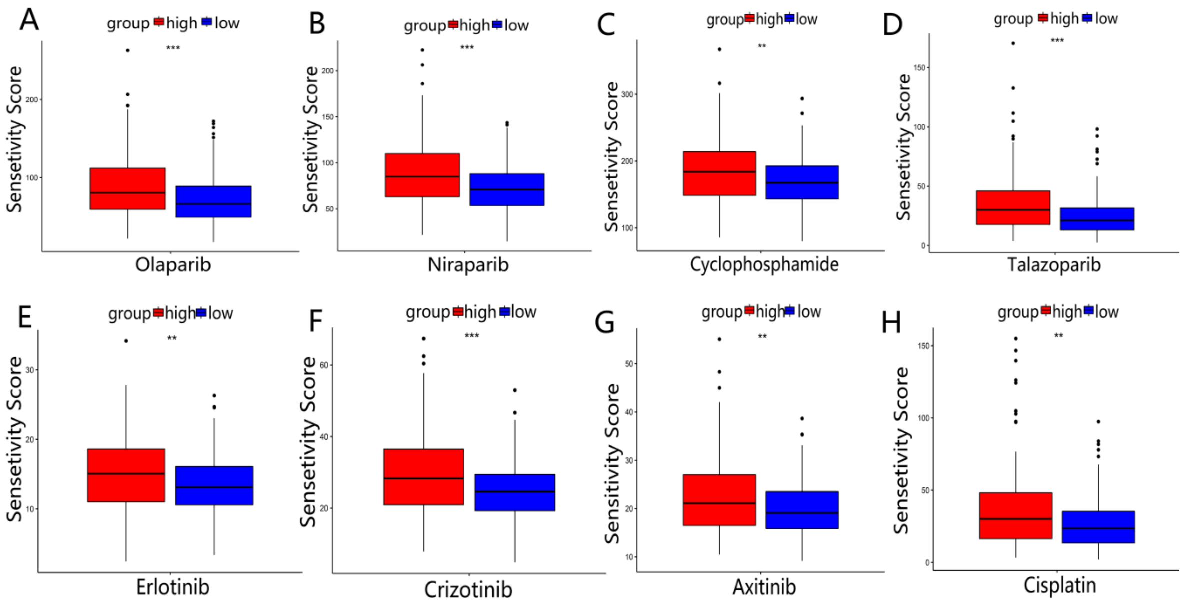

Figure S3. Drug sensitivity analysis in GSE20685-BRCA. **(A–D)** The sensitivity analysis of 4 BRCA common chemotherapy agents (Olaparib, Niraparib, Cyclophosphamide, and Talazoparib) in two risk groups. **(E–H)** The sensitivity analysis of 4 other cancers's common chemotherapy agents (Erlotinib, Crizotinib, Axitinib, and Cisplatin) for two risk groups. * $p < 0.05$; ** $p < 0.01$; *** $p < 0.001$.

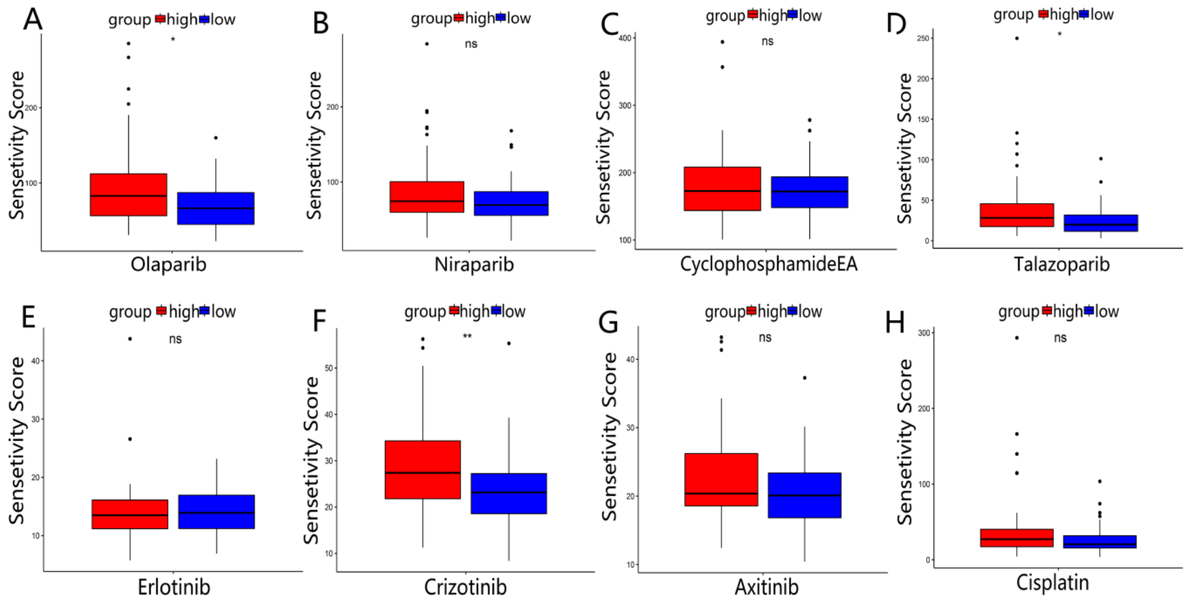

Figure S4. Drug sensitivity analysis in GSE88770-BRCA. **(A–D)** The sensitivity analysis of 4 BRCA common chemotherapy agents (Olaparib, Niraparib, Cyclophosphamide, and Talazoparib) in two risk groups. **(E–H)** The sensitivity analysis of 4 other cancers's common chemotherapy agents (Erlotinib, Crizotinib, Axitinib, and Cisplatin) for two risk groups. * $p < 0.05$; ** $p < 0.01$; *** $p < 0.001$.
