# Supplementary material for: Identification of a Novel Gene Signature with DDR and EMT Difunctionalities for Predicting Prognosis, Immune Activity, and Drug Response in Breast Cancer
Source: Int J Environ Res Public Health. 2023 Jan 10;20(2):1221. doi: 10.3390/ijerph20021221 (PMC9859620; doi:10.3390/ijerph20021221)
Supplement: Supplementary file 1 [file ijerph-20-01221-s001.zip › Supplementary file S9.pdf]

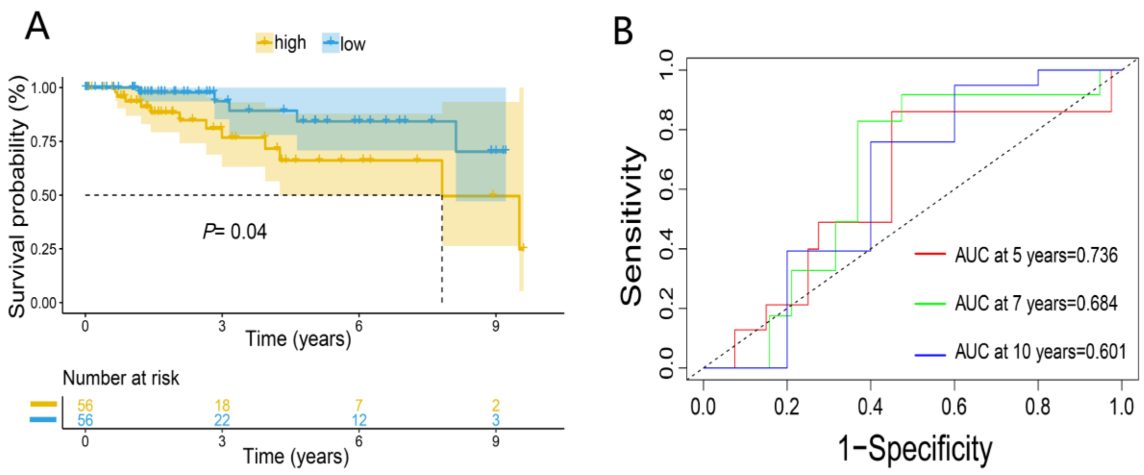

Figure S1. Survival analysis and ROC curves for 112 patients with triple-negative TCGA-BRCA. (**A**) Survival analysis for 112 patients with triple-negative TCGA-BRCA. (**B**) ROC curves for 112 patients with triple-negative TCGA-BRCA.

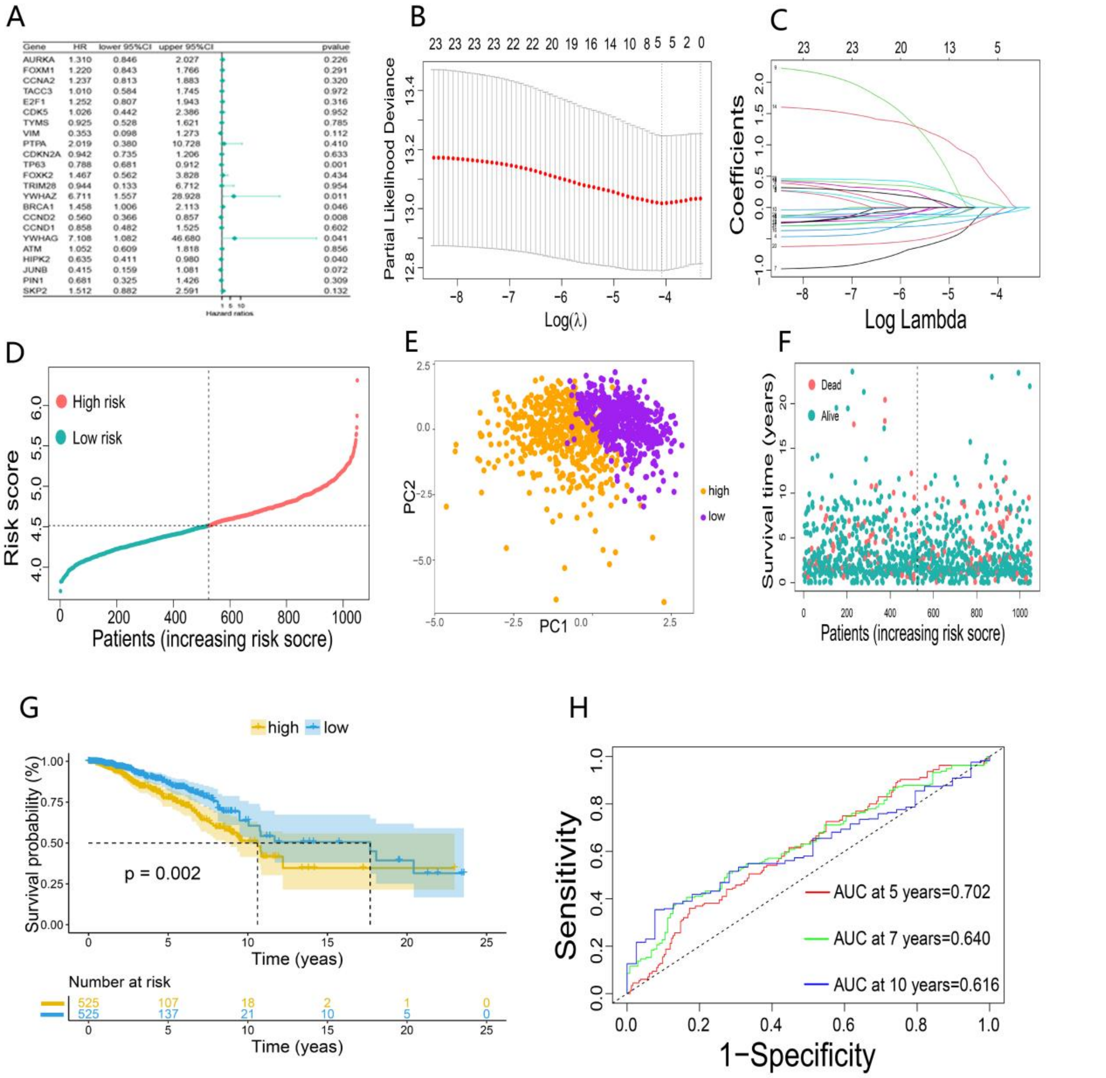

Figure S2. Establishment of 23-DEDGs prognostic model based on the TCGA cohort. **(A)** Forest plot of the univariate Cox regression for 23 DEDGs. **(B)** Cross-validation for optimal parameter selection in the LASSO regression. **(C)** LASSO regression for 5 OS-related DEDGs. **(D)** Distribution of patients based on the risk score. **(E)** Principal component analysis (PCA) of 5 DEDGs. **(F-G)** The survival status and overall survival analysis of the two risk groups. **(H)** The time-dependent receiver operating characteristic (ROC) curve of 5 DEDGs.

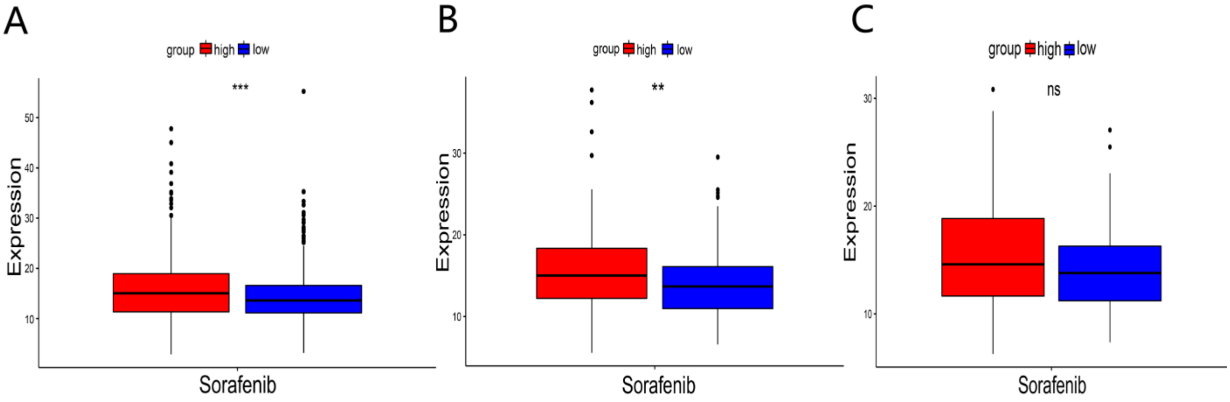

Figure S3. Sensitivity analysis of EMT-targeted drug "Sorafenib" in TCGA and GEO databases. (**A**) Sensitivity analysis of EMT-targeted agent "Sorafenib" in TCGA-BRCA. (**B**) Sensitivity analysis of EMT-targeted agent "Sorafenib" in GSE20685-BRCA. (**C**) Sensitivity analysis of EMT-targeted agent "Sorafenib" in GSE88770-BRCA. * $p < 0.05$; ** $p < 0.01$; *** $p < 0.001$.
